# The structure of cognitive strategies for wayfinding decisions

**DOI:** 10.1007/s00426-023-01863-3

**Published:** 2023-08-09

**Authors:** Otmar Bock, Ju-Yi Huang, Oezguer A. Onur, Daniel Memmert

**Affiliations:** 1 Institute of Exercise Training and Sport Informatics, German Sport University, Cologne, Germany; 2 Department of Neurology, Faculty of Medicine and University Hospital Cologne, University of Cologne, Cologne, Germany

## Abstract

Literature proposes five distinct cognitive strategies for wayfinding decisions at intersections. Our study investigates whether those strategies rely on a generalized decision-making process, on two frame-specific processes—one in an egocentric and the other in an allocentric spatial reference frame, and/or on five strategy-specific processes. Participants took six trips along a prescribed route through five virtual mazes, each designed for decision-making by a particular strategy. We found that wayfinding accuracy on trips through a given maze correlated significantly with the accuracy on trips through another maze that was designed for a different reference frame (*r*_between-frames_ = 0.20). Correlations were not significantly higher if the other maze was designed for the same reference frame (*r*_within-frames_ = 0.19). However, correlations between trips through the same maze were significantly higher than those between trips through different mazes that were designed for the same reference frame (*r*_within-maze_ = 0.52). We conclude that wayfinding decisions were based on a generalized cognitive process, as well as on strategy-specific processes, while the role of frame-specific processes—if any—was relatively smaller. Thus, the well-established dichotomy of egocentric versus allocentric spatial representations did not translate into a similar, observable dichotomy of decision-making.

## Introduction

Finding our way through a building or city requires a range of sensory, cognitive, and motor functions. We need to process spatial information from multiple sensory modalities, maintain internal representations of the environment, plan routes, make decisions at intersections, control our gait, monitor our position and heading in space, and orchestrate these processes by overarching executive control (review, e.g., in Hegarty et al., 2022; Wolbers & Hegarty, 2010). The present study deals specifically with decision-making at intersections.^1^ Consider, e.g., a conference attendee who walks from the hotel to the convention center. This person will eventually arrive at an intersection and must decide whether to walk straight on, turn left, or turn right. The traveler will then proceed in the chosen direction towards the next intersection, decide again, etc. This will continue until the attendee arrives at the convention center—or goes astray if one or more direction choices are inadequate. Thus, successful wayfinding involves a sequence of adequate direction choices.

It has been proposed that travelers use different cognitive strategies for decision-making at intersections. With the *serial order strategy* (Iglói et al., 2009; Tlauka & Wilson, 1994), they recall a sequence of directions to take, such as “first left, then straight, then right”. With the *associative cue strategy* (Tlauka & Wilson, 1994; Waller & Lippa, 2007), they recall associations between local landmarks and directions, such as “at the drug store turn right”. Local landmarks can be any natural or man-made objects which are visible near intersections, where they stand out because of their distinctive physical properties, or because of their symbolic or emotional meaning (review, e.g., in Yesiltepe et al., 2021). With the *beacon strategy* (Waller & Lippa, 2007), travelers select directions that bring them closer to a visible landmark, either to a local landmark that is visible only from nearby, or a global landmark that is visible from many places, such as a TV tower. With the *relative location strategy* (Jacobs et al., 1997; Morris, 1984), travelers select directions that bring them closer to a location inferred from multiple global landmarks, e.g., to a destination located mid-between a TV tower and a conspicuous high-rise building. Finally, with the *cognitive map strategy* (O’Keefe & Nadel, 1978; Tolman, 1948), travelers base their decisions on an internal representation of the environment, and of their own position and heading within that environment. For example, a traveler who knows to be in Trafalgar Square facing east can deduce from a cognitive map of central London that a right turn is needed to reach Westminster Abbey.

Many wayfinding tasks of everyday life can be completed successfully by more than one decision-making strategy. Thus, the conference attendee in the above example can reach the convention center by replicating a series of directions, by associating local landmarks with directions, by walking successively closer towards a location inferred from one or multiple global landmarks (if available), or by perusing a cognitive map of the convention city. Which of those strategies the traveler selects seems to depend on factors such as environmental topography, earlier experience, and individual preferences (Hölscher et al., 2009; Iaria et al., 2003; Wiener et al., 2009). It has been proposed that travelers even can change their decision strategy in the middle of a given trip (Hamburger, 2020; Wolbers & Hegarty, 2010).

Wayfinding literature hypothesized that decision-making strategies fall into two categories. One category is formed by response strategies, which encode actions in an egocentric reference frame, that is, relative to one’s own momentary position and heading; the other category is formed by place strategies, which encode the spatial configuration of the environment in an allocentric reference frame, that is, independent of one’s own momentary position and heading (e.g., Hegarty et al., 2022; Marchette et al., 2011). Single-cell, lesion and neuroimaging studies indeed provide converging evidence for such a dichotomy, with response strategies relying on parieto-striatal circuitry and place strategies relying on the hippocampus (review, e.g., in Chersi & Burgess, 2015; Hegarty et al., 2022). Based on this evidence, it is conceivable that the serial order, associative cue, and beacon strategy are response strategies that rely on a common, parieto-striatal circuitry, while the relative location and the cognitive map strategy are place strategies that rely on the hippocampus.

However, the notion of two distinct decision-making processes is not the only conceivable view. Wayfinding decisions rely not only on spatial representations but also on cognitive skills which are not coded in two distinct reference frames, such as visuo-spatial attention, working memory, and executive functions. Accordingly, it has been shown that wayfinding-related brain activity in the retrosplenial and in the medial prefrontal cortex occurs both during egocentric and during allocentric tasks (Doeller et al., 2008; Ino et al., 2002; Latini-Corazzini et al., 2010). These and/or other brain areas might, therefore, provide the neural substrate for generalized, frame-independent processes that support all five above decision-making strategies mentioned above.

Finally, it should also be considered that each strategy has its own specific cognitive demands, such as the identification of local or global landmarks, the memory for items versus for associations between items, and the internal representation of two objects versus of urban layouts. Such strategy-specific cognitive demands might well give rise to strategy-specific decision processes.

The purpose of the present study is to provide experimental evidence for or against the existence of one generalized, two frame-specific, and/or five strategy-specific decision-making processes. To this end, we designed five virtual mazes, each requiring a different strategy: Maze S called for the serial order strategy, maze A for the associative cue strategy, maze B for the beacon strategy, maze R for the relative location strategy, and maze C for the cognitive map strategy. Each participant took six trips through all mazes. The first trip through a given maze was externally guided, and served to demonstrate the required strategy. The subsequent five trips were self-guided, and served to quantify participants’ proficiency in using that strategy. We reasoned that, if a generalized decision process exists, then participants who perform particularly well on a given trip should tend to perform particularly well on all other trips, even on trips through other mazes that require decisions in a different reference frame. Thus, participants’ performance on trips through mazes with different reference frames should be correlated. We further reasoned that, if two frame-specific decision processes exist, then participants’ performance on trips through mazes with the same reference frame should be more closely correlated than on trips through mazes with different reference frames. We finally reasoned that, if strategy-specific decision processes exist, then participants’ performance on trips through the same maze should be more closely correlated than on trips through different mazes, even through mazes with the same reference frame.

We also were interested to determine whether participants established an internal spatial representation of the route they traveled. Such a representation could be relevant for performance on maze C, but would be only incidental for the other mazes. To find out, we asked participants to indicate the end-to-start direction of the last route traveled in each maze.

## Methods

### Participants

Thirty volunteers (26.0 ± 2.8 years of age, 20 females) were examined. They were healthy by self-report, and those who wore eyeglasses in everyday life continued to wear them in testing. The highest education level was a high school exam for *n* = 7, a bachelor’s degree for *n* = 16, and a master’s degree for *n* = 7. The study was conducted in adherence to the Helsinki declaration. It was part of a research program approved by the Ethics Commission of the German Sport University. All participants signed an informed consent statement before testing began.

### Decision-making task

The participants were asked to follow a prescribed route through a virtual maze that was presented on a computer screen. They were transported passively to an intersection of corridors, where they stopped. Then they had 3000 ms to indicate whether the route continued straight on, to the left, or the right, by deflecting the handle of a joystick in the pertinent direction. They could respond anytime during the 3000-ms interval, ad were not rushed to do this quickly. During the subsequent 500 ms, participants received feedback on whether their response was correct (green ‘o’), incorrect (red ‘x’) or missing (red ‘?’). Premature handle deflections, executed before stopping at an intersection, were ignored by the software. After the feedback, participants were transported passively in the correct direction along the prescribed route, irrespective of the response they gave. Transport occurred at a constant speed and took 2000 ms, at which time participants arrived at the next intersection and stopped. Thus, the total time from one intersection to the next was 3000 ms (response interval) + 500 ms (feedback interval) + 2000 ms (transport) = 5500 ms. This fixed timing was implemented since the decision-making task was designed for future use with EEG registrations. Stimulus display and response recording were controlled by custom software using Presentation^®^ (Version 22.1, Neurobehavioral Systems, Inc., Berkeley, CA, USA).

Each participant took six trips through a given maze. The first trip was externally guided: an arrow was displayed on the maze floor throughout the response interval, indicating the direction in which the route continued. The subsequent five trips through that maze were self-guided: no arrows were displayed, and participants had to decide on their own where the route continued. A shining trophy was displayed at the end of each trip as a virtual reward. Once all six trips through a given maze were completed, the next maze followed after a brief rest break, for a total of five different mazes. Thus, participants had to take 6 (trips per maze) * 5 (mazes) = 30 trips altogether.

Performance was quantified as response accuracy, i.e., the proportion of correct responses per trip. It could range from 0 = all responses were wrong to 1 = all responses were correct. Total trip time was not analyzed, since this quantity was fixed (see above). Reaction time (arrival at the intersection to onset of response) was not analyzed either, since this quantity had a different meaning in different mazes. In some mazes, participants could decide which way to proceed even before reaching an intersection, and reaction time therefore mainly represented motor delays. In other mazes, however, participants could make their decision only after reaching an intersection, and reaction time, therefore, comprised of both decision-making and motor delays.

### Strategy-specific mazes

**Fig. 1:**
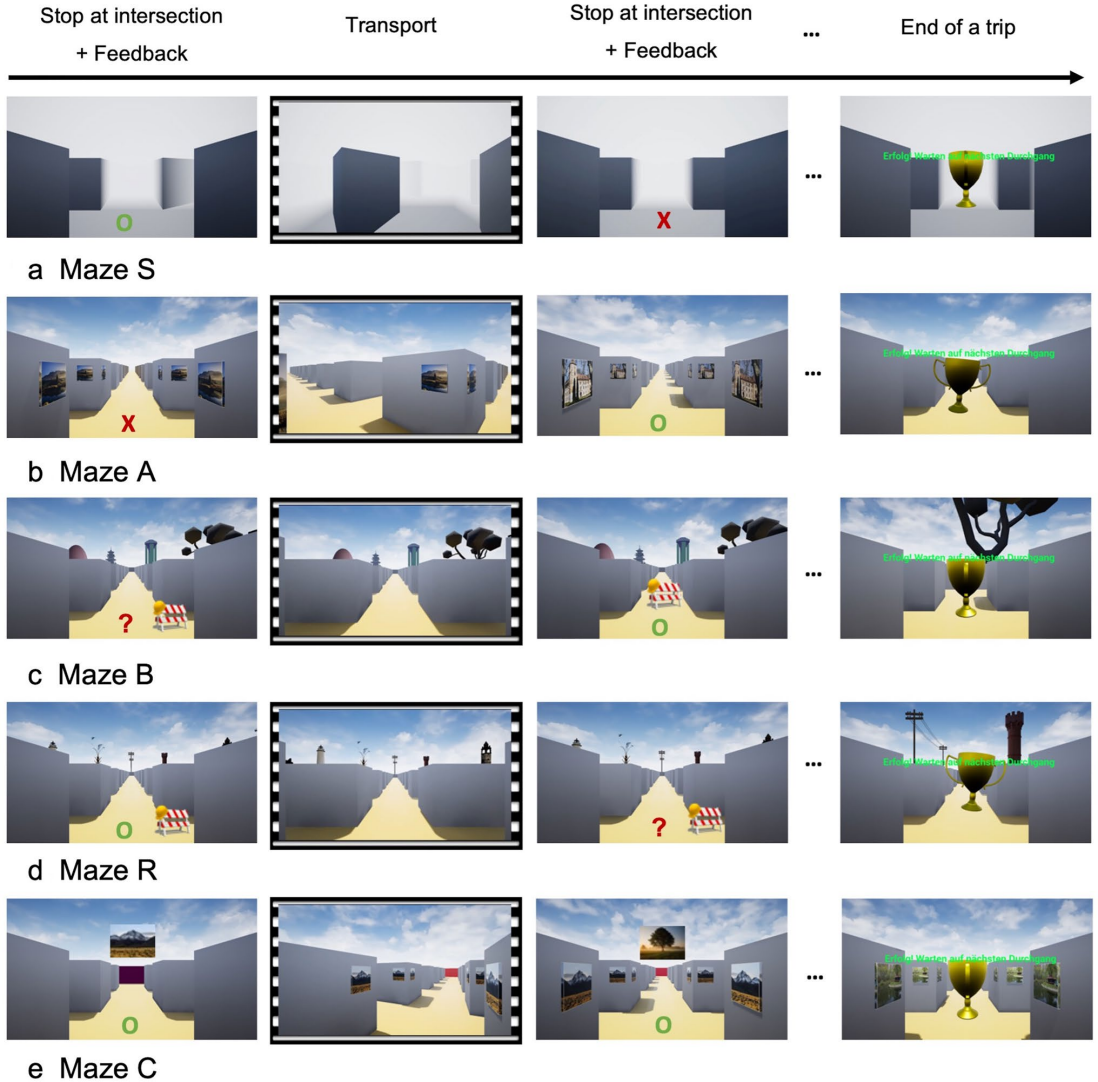
Exemplary snapshots from a trip through each maze. Stops at intersections show the feedback symbols, indicating a correct response (green ‘o’), incorrect response (red ‘x’), or no response (red ‘?’). Only the feedback interval, not the preceding response interval is shown. Sprocket holes symbolize passive transport through the maze. Note the oblique departure of the right-side corridor at the first intersection of maze S. Note also that the external wall of maze C is purple at the first but red at the second intersection, as the participant looks in another direction (color figure online)

Maze S was designed for decision-making by the *s*erial order strategy. As in earlier research about this strategy (e.g., Tlauka & Wilson, 1994), this maze consisted of uniform corridors without global or local landmarks (see Fig. 1a). Participants were instructed to follow the same route across twelve intersections on all six trips; since landmarks were absent, they could only succeed by recalling the sequence of twelve directions they had to take. Notably, some intersections had corridors departing to the left or right at an angle of 60° rather than the usual 90°. The number of those intersections (none, one, or two), and their location along the route, varied from trip to trip, such that the shape of the route through allocentric space was different on each trip. This was to discourage participants from forming a cognitive map of the route.

Maze A was designed for the *a*ssociative cue strategy. In this and all subsequent mazes, corridors always departed at 90° angles. Local landmarks were presented at all intersections; they were photographs of natural scenes or historical buildings. The same local landmark was displayed at all corners of a given intersection, but different photographs were posted at different intersections (see Fig. 1b). They became visible only at the onset of the response interval. Participants were again instructed to follow the same route across twelve intersections on all six trips; they were told that the landmarks will help them to find the way. As in earlier research about the associative cue strategy (Bock & Borisova, 2022), the serial order of landmarks varied from trip to trip, but the direction associated with each landmark remained fixed on all trips. For example, a castle was shown at the second intersection of the first trip, and the same castle was shown again at the eighth intersection of the second trip; irrespective of this changing order, however, the correct response at that castle was always to proceed straight on. Since the order of landmarks and directions varied from trip to trip, participants could succeed by recalling landmark–direction associations, but not by recalling the serial order of directions. The mixed order of directions also ensured that the allocentric shape of the route was different on each trip, which again discouraged the formation of a cognitive map.

Maze B was designed for the *b*eacon strategy. Thirteen global landmarks were presented equidistantly around the circumference of a 15 × 15 grid maze; they were natural or architectural 3D objects, high enough to be seen from anywhere within the maze. The landmarks remained visible throughout each trip (see Fig. 1c), but the landmark array was shifted en bloc around the maze after each trip. Thus, landmarks changed their position unpredictably from trip to trip, while maintaining their neighborhood relations. Participants were instructed to go on each trip along the shortest possible route to one particular landmark, a conspicuous tree. Since several shortest possible routes existed, one of them was enforced by placing barriers at the entrance to alternative routes. For example, a barrier at the entrance to a left corridor required participants to go first straight, then left, rather than going first left, then right to reach the same location. This ensured that visual stimulation and motor responses were the same for all participants when our software is used for EEG registrations. The starting point of each trip was set such that the shortest possible route always comprised twelve intersections.

Since the tree in maze B changed its position between trips, the number of intersections varied from trip to trip, as did the required sequence of directions. This rendered the serial order strategy ineffective. Since the tree was not associated with a fixed direction at all intersections and all trips, the associative cue strategy was ineffective as well. Participants could form a cognitive map of the environment, with global landmarks that change their allocentric locations after each trip, but such a map was not needed to go to the visible tree.

Maze R was designed for the *r*elative location strategy. It differed from maze B only in that a different set of thirteen global landmarks was used, and that participants were instructed to go to an invisible destination which formed an equilateral triangle with two adjacent landmarks, a medieval tower, and a set of power poles (see Fig. 1d). We explained beforehand that the distance between the two landmarks equaled the distance between the invisible destination and either landmark. Again, participants had to take the shortest possible route, and one particular shortest possible route was enforced by barriers. Again, the landmark array was shifted en bloc after each trip, and the serial order strategy as well as the associative cue strategy were ineffective. Again, participants could form a cognitive map, with global landmarks that change their allocentric locations after each trip, but such a map was not needed to reach the location that was equidistant to the visible tower and poles.

Maze C was designed for the *c*ognitive map strategy. Twelve local landmarks were presented at every second intersection of a 7 × 9 grid maze. They were different photographs than those in maze A, but again, the same photograph was posted at all corners of a given intersection, different photographs appeared at different intersections, and they became visible only at the onset of the response interval. The allocentric locations of the landmarks remained fixed across all trips. Participants were instructed to walk towards one of the landmarks by displaying that landmark as a floating picture above the maze throughout the response and feedback interval (see Fig. 1e). When participants stopped at the instructed intersection, that landmark became visible on the edges of the wall, and a new instructed landmark was displayed as floating above the maze. This was repeated until all twelve landmarks have been visited. Again, participants were asked to take the shortest possible route. If more than one shortest possible route existed, which was only the case before reaching the first landmark, they had to take the route that continued straight on. Barriers therefore were not needed. Notably, the order of instructed landmarks differed from trip to trip; hence the order of directions to take differed as well, as did the associations between landmarks and directions. The serial order strategy and the associative cue strategy, thus, were ineffective. Participants could only succeed by forming a cognitive map of the maze with its landmark array, and of their own allocentric position and heading in the maze. To facilitate the awareness of heading, the four external walls of the maze were painted in four different colors.

Since mazes A and C presented local landmarks at intersections, participants could decide which way to proceed only after reaching the pertinent intersection. Mazes B and R used continuously visible global landmarks, but participants could still decide only after reaching the intersection because they could not anticipate the location of barriers. However, mazes B and R allowed participants to prepare for two alternative responses, while mazes A and C involved three alternative responses. Finally, maze S presented no landmarks and allowed participants to decide well ahead of time, even several intersections in advance. It, therefore, is conceivable that the mazes differed not only with respect to the type of strategy required, but also with respect to the diffculty for implementing that strategy.

### Procedures and data analysis

The five mazes were administered to participants in a balanced order, according to a latin square, using the instructions listed in the Appendix. After the last trip in each maze, participants were asked to indicate the starting location of that trip with respect to their final position and heading. For this, they were given a sheet of paper with a sketch of (their) head and were asked to draw a line from the center of that head toward the starting location. The angular difference between the drawn line and the actual direction to the starting location was recorded as a pointing error. The total time to complete all mazes and pointing tasks were about 80 min.

Once the data were collected, we calculated the bivariate correlations between participants’ performance on all self-guided trips, i.e., on 5 (self-guided trips per maze) * 5 (mazes) = 25 trips. These correlations were grouped into

- *r*_within-maze_: correlations between trips through the same maze
- *r*_within-frame_: correlations between trips through different mazes with the same reference frame
- *r*_between-frames_: correlations between trips through different mazes with different reference frames.

The *r*-scores were transformed to Fisher’s *Z* to ensure normal distribution and were then submitted to *t*-tests for two samples with different variances, or to *t*-tests of a single sample against a reference value of zero. The *t*-tests compared the scores from three different correlation groups in three different data sets, each with a different number of scores, as shown in Table 2. Required sample size was calculated by G*Power (Faul et al., 2007) for each comparision, using *α* = 0.05, 1 − *ß* = 0.8, *d* = 0.5, and the respective number or ratio of scores. We, thus, yielded for data set 1 a required sample size of 80, 96 and 48 for *r*_between-frames_, *r*_within-frame_, and *r*_within-maze_, respectively, which is less than the actually analyzed sample sizes (cf. Table 2). For sets 2 and 3, however, required sample sizes wer higher than the actually analyzed sample sizes. The chance of type a II error, therefore, exceeded the predetermined acceptance threshold, hence non-significant fidings must be interpreted with caution. Note that the sample size of our dependent variable is determined by our experimental design—specifically, by the number of trials in each maze (cf. Table 1)—and cannot be increased by testing more participants.

**Table 1:**
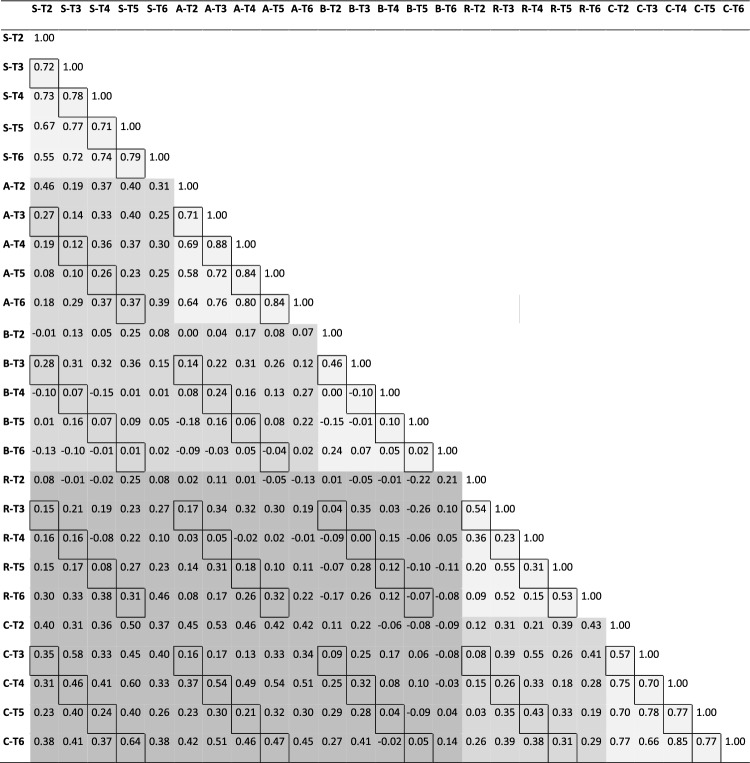
Bivariate correlations between participants’ accuracy on different trips

S, A, B, R, and C represent the five strategy-specific mazes, and T2–T6 represent the five self-guided trips. For example, C-T6 is the last self-guided trip in maze C. Shadings indicate correlations between trips from the same maze (light gray), from different mazes with the same reference frame (middle gray), and from different mazes with different reference frames (dark gray), respectively. Framed cells represent correlations that are mutually independent

Pointing errors were analyzed using the R package *CircStats* (Jammalamadaka & SenGupta, 2001). We calculated for each maze the mean pointing error across participants with the function ‘circ.mean’, and the concentration of errors about that mean with the function ‘circ.disp’. We further conducted Rao’s test for the uniformity of distributions with the function ‘rao.spacing’, and Watson’s test of two-sample homogeneity—comparing maze C to each of the other mazes—with the function ‘watson.two’.

## Results

**Fig. 2:**
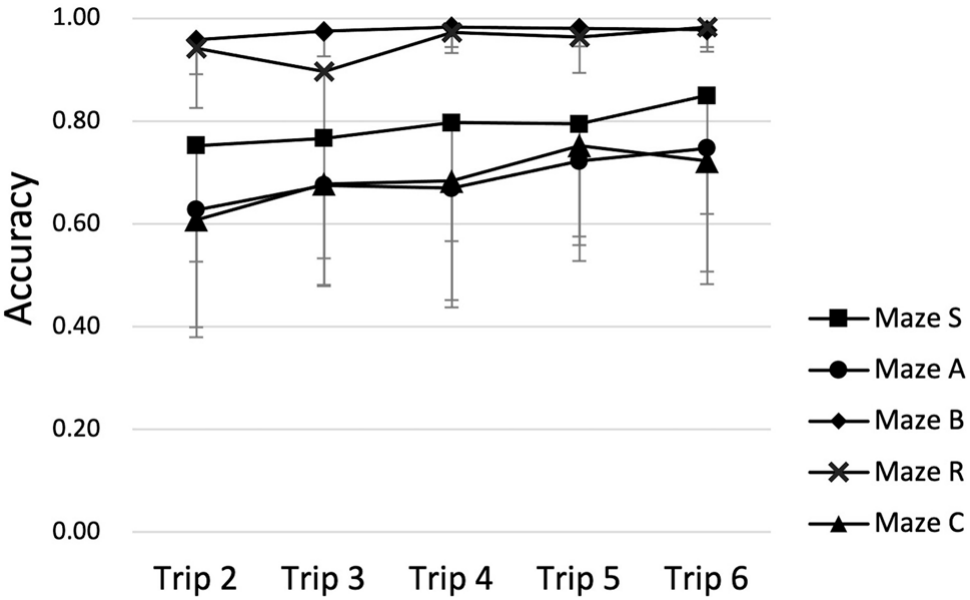
Mean accuracy in each maze on all self-guided trips. Symbols indicate the across-individual means accuracy, and error bars indicate the pertinent standard deviations

**Fig. 3:**
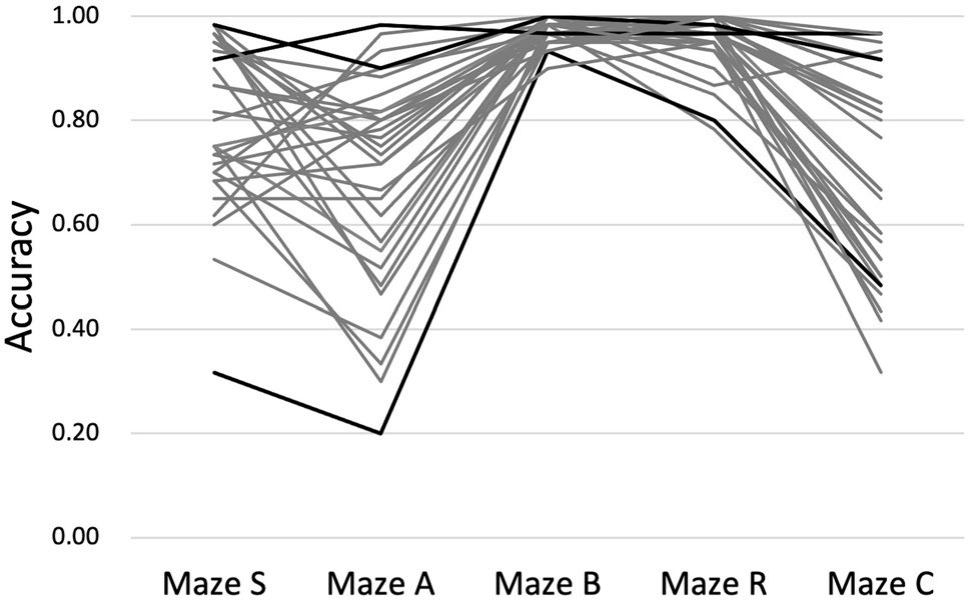
Mean accuracy of each individual on all self-guided trips. Each line represents one person. Bold lines indicate the two persons with the highest and the person with the lowest mean accuracy

Figure 2 illustrates that the mean accuracy across participants was higher in mazes B and R than in the other three mazes, and that it gradually increased from trip to trip. Figure 3 shows that the mean accuracy across trips varied considerably between participants, less so in mazes B and R than in the other three mazes. It also shows that some participants tended to be consistently better or worse than others.

Table 1 indicates the bivariate correlations between participants’ accuracy on all self-guided trips in all mazes. Cells in light gray represent *r*_within-maze_, those in middle gray represent *r*_within-frame_, and those in dark gray represent *r*_between-frames_. Descriptive statistics for these three groups of correlations are provided in a row ‘Set 1’ of Table 2. *t*-tests yielded that

- *r*_between-frames_ is significantly larger than zero (*t*(149) = 12.71; *p* < 0.001),
- *r*_within-frame_ is not significantly different from *r*_between-frames_ (*t*(239.96) = − 0.84; *p* = 0.401), and
- *r*_within-maze_ is significantly larger than *r*_within-frame_ (*t*(56.90) = 7.66; *p* < 0.001).

**Table 2:** Descriptive statistics for the three different groups of correlations

	*r*_within-maze_	*r*_within-frame_	*r*_between-frames_
Set 1: mean ± SD (*n*)	0.52 ± 0.30 (50)	0.19 ± 0.16 (100)	0.20 ± 0.19 (150)
Set 2: mean ± SD (*n*)	0.56 ± 0.29 (20)	0.18 ± 0.13 (16)	0.21 ± 0.18 (24)
Set 3: mean ± SD (*n*)	0.76 ± 0.08 (12)	0.26 ± 0.09 (4)	0.38 ± 0.16 (8)

Set 1 refers to all correlations in Table 1, set 2 only to mutually independent correlations, and set 3 only to mutually independent correlations from mazes S, A, and C

One could argue that the above *t*-tests might be biased since the correlations in Table 1 violate the *t*-test prerequisite of independence. For example, if the correlation between some variables A and B is high positive, and the correlation between variables B and C is high positive as well, then the correlation between A and C is bound to be at least moderately positive, and therefore is not independent of the other two correlations. To avoid this problem, we repeated the *t*-tests with a reduced data set that did not include such recursive correlations; this reduced data set corresponds to the framed cells in Table 1, and its descriptive statistics are included as row ‘Set 2’ in Table 2. The new *t*-tests yielded, as with the full data set, that

- *r*_between-frames_ is significantly larger than zero (*t*(23) = 5.36; *p* < 0.001),
- *r*_within-frame_ is not significantly different from *r*_between-frames_ (*t*(37.82) = − 0.62; *p* = 0.538), and
- *r*_within-maze_ is significantly larger than *r*_within-frame_ (*t*(24.24) = 5.20; *p* < 0.001).

One could further argue that the latter *t*-tests might still be inadequate since the data from mazes B and R indicate that those two mazes require little if any decision-making (see “Discussion” for more details). We, therefore, repeated our analyses with an even more reduced data set that excluded trips registered in mazes B and R. Descriptive statistics are included as row ‘Set 3’ in Table 2. Again, *t*-tests yielded that

- *r*_between-frames_ is significantly larger than zero (*t*(7) = 6.30; *p* < 0.001),
- *r*_within-frame_ is not significantly different from *r*_between-frames_ (*t*(9.40) = − 1.59; *p* = 0.146), and
- *r*_within-maze_ is significantly larger than *r*_within-frame_ (*t*(4.38) = 8.84; *p* < 0.001).

Excluding mazes B and R led to a substantial increase in the magnitude of correlations (cf. Table 2, ‘Set 3’). Conversely, the magnitude of correlations for B and R alone was very low, with *r*_within-maze_ = 0.26 ± 0.24 and *r*_between-frames_ = 0.02 ± 0.08.

**Fig. 4:**
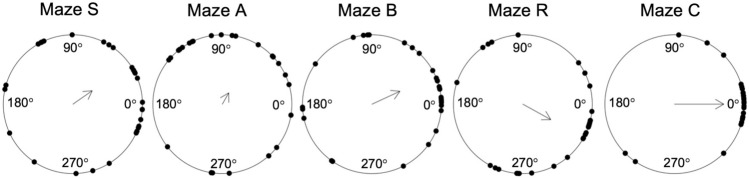
Outcome of the pointing test. Dots represent the pointing errors of different persons, and arrows are the resultant vectors; arrow direction indicates the mean error, and arrow length indicates data concentration about that mean (length = 1: perfectly aligned data; length = 0: data scattered all around the circle)

Figure 4 illustrates the outcome of the pointing test that was administered after the last trip in each maze. It shows that the pointing errors were not randomly scattered throughout the full 360° range, but rather were concentrated about a mean error of less than ± 90°. The concentration was highest, and the mean error was smallest, after maze C. In accordance with these observations, Rao’s test yielded a significant deviation from uniformity after all mazes (all *U* > 168, all *n* = 30, all *p* < 0.05). Furthermore, Watson’s test revealed that the distribution of pointing errors after maze C differed significantly from that after maze S (*U*^2^ = 0.29, *n* = 30, *p* < 0.01), maze A (*U*^2^ = 0.47, *n* = 30, *p* < 0.001), and maze R (*U*^2^ = 0.29, *n* = 30, *p* < 0.001), but not after maze B (*U*^2^ = 0.15, *n* = 30, *p* > 0.10).

The participants’ absolute pointing errors after maze C were significantly correlated with their mean accuracy in that maze (*r* = − 0.46; *p* = 0.011). The negative sign of *r* indicates that a higher accuracy in maze C was associated with a lower pointing error. The correlation between maze accuracy and pointing errors was negative without reaching significance in maze S (*r* = − 0.33; *p* = 0.074), maze R (*r* = − 0.31; *p* = 0.095), and maze A (*r* = − 0.26; *p* = 0.158), but it was negligible in maze B (*r* = − 0.06; *p* = 0.752).

## Discussion

The present study dealt with the decision-making component of wayfinding. Literature suggests that travelers decide which way to proceed across an intersection by using five possible cognitive strategies, and we wanted to find out whether those strategies are implemented by a single, generalized decision-making process, by two frame-specific processes—one operating in an egocentric and the other in an allocentric reference frame, and/or by five distinct strategy-specific processes. To find out, we designed five strategy-specific mazes and analyzed the correlations between participants’ decision accuracy on repeated trips through those mazes.

A first look at the registered data revealed that participants were much more accurate, and exhibited much less interindividual variability, in mazes B and R than in the other three mazes. We have two possible interpretations for this observation. First, mazes B and R required decisions between two rather than three response alternatives, and thus were cognitively less demanding than the other mazes (see, e.g., Damos & Wickens, 1977). Second, mazes B and R implemented decision-making strategies that per se were less demanding, irrespective of the number of alternatives: participants simply had to orient towards one or two permanently visible landmarks, which probably required only limited cognitive processing, in particular little decision-making.

We found that participants’ accuracy on trips through a given maze correlated significantly with their accuracy on trips through another maze, even if that other maze was designed for a strategy in a different reference frame. Although correlations were only modest in magnitude (*r*_between-frames_ = 0.20), they nevertheless suggest the existence of a generalized, frame-independent process. This process might reflect core cognitive skills which are not coded in two distinct reference frames, such as visuo-spatial attention, working memory, and executive functions (cf. “Introduction”). The neural substrate for such a process could be in the retrosplenial and/or in the medial prefrontal cortex, where activation was observed during both egocentric and allocentric tasks (Doeller et al., 2008; Ino et al., 2002; Latini-Corazzini et al., 2010).

We further found that the correlations were not significantly higher when the other maze involved the same rather than a different reference frame; in fact, those correlations even were minimally smaller (*r*_within-frame_ = 0.19). We, thus, found no support for the existence of frame-specific processes. This negative outcome does not imply that there are no such processes; on the contrary, behavioral and neurophysiological data provide compelling evidence that our brain indeed encodes spatial information both in an egocentric and in an allocentric reference frame (review, e.g., in Chersi & Burgess, 2015; Colombo et al., 2017; Hegarty et al., 2022). However, the dichotomy between egocentric and allocentric representations of space apparently did not translate into an observable dichotomy between egocentric and allocentric decision-making. The role of frame-specific decision processes, therefore, might be small or absent.

Finally, we found that the correlations between repeated trips through the same maze were quite substantial (*r*_within-maze_ = 0.52). They were significantly higher than the correlations between trips through different mazes, even if those mazes involved the same reference frame. This supports the view that wayfinding decisions are made by strategy-specific processes. It remains open at present what the neural substrate of those processes might be. It also remains open whether the number of strategy-specific processes is limited to five: our study dealt with the five decision strategies described in the extant literature, but additional strategies might exist.

Notably, the above pattern of findings was yielded not only with the full data set of correlations, but also with a substantially reduced data set that excluded potential interdependence of scores, and even with an even more reduced data set limited to mazes S, A, and C. This replicability attests to the robustness of the observed pattern, although the non-significant difference between *r*_within-frame_ and *r*_between-frames_ in the reduced data sets must be interpreted with caution (see “Methods”). However, the consistent emergence—in all three data sets—of non-significant differences between *r*_within-frame_ and *r*_between-frames_ highly significant differences between *r*_within-maze_ and *r*_within-frame_, and highly significant differences between *r*_between-frames_ and zero (all *p* > 0.001), strengthens our conficdence that the role of frame-specific decision processes is small compared to that of frame-independent processes, and that of strategy-specific processes.

Furthermore, *r*_within-maze_ as well as *r*_within-frame_ and *r*_between-frames_ were substantially smaller in mazes B and R than in mazes S, A, and C, which provides additional credibility to the above assumption that mazes B and R required little, if any, wayfinding and decision-making skills. Specifically, the latter two mazes may not be part of the generalized process proposed above, since their *r*_between-frames_ was as low as 0.02.

After their last trip through each maze, participants were asked to point in the direction where that trip started. Their responses were not randomly scattered throughout the full 360° range, but rather were concentrated in the correct hemispace (i.e., mean pointing error was less than ± 90°), which suggests that participants established at least a coarse spatial representation of their last trip. Interestingly, this was the case even in mazes S, A, B and R, which did not require a spatial representation for successful wayfinding. In those mazes, therefore, a coarse spatial representation was formed incidentally—with no bearing on maze decisions—or it was formed in support of decision-making. Such support would be a manifestation of dual encoding, in that decisions were based not only on the intended strategy, but also on the presumed spatial representation. It has indeed been documented before that dual encoding enhances cognitive performance (Paivio & Csapo, 1973), also in route decision tasks (Bock et al., 2023).

Pointing errors were smaller in maze C than for any other maze, and were significantly correlated with maze accuracy only in maze C. We attribute this pattern of findings to the fact that maze C not only *allowed* participants to establish a spatial representation, it rather was the only maze that actually *required* them to establish a detailed representation to perform successfully. We, therefore, assume that participants used this detailed spatial representation to accomplish not only the maze task but also the pointing task. This could explain both the low pointing errors in maze C and their correlation with maze accuracy.

Compared to maze C, pointing errors were somewhat but not significantly larger in maze B, and their correlation with maze accuracy was nearly zero. We attribute these findings to the fact that maze B required little if any decision-making (see above), thus leaving most of the participants’ cognitive resources available to establish a moderately detailed but task-irrelevant spatial representation. This could explain why pointing performance was relatively good, yet was poorly correlated with maze accuracy.

In conclusion, our data suggest that wayfinding decisions are initiated by multiple strategy-specific processes, as well as by a generalized, strategy-independent process. If it is necessary for correct decisions, a detailed spatial representation is formed; otherwise, a coarse spatial representation is formed. Since these representations emerge in all mazes, they could well be related to the surmised strategy-independent decision process.

## Data Availability

Data are available from the corresponding author upon reasonable request.

## Appendix: Participants’ instructions

At the onset of the experiment: “In the following tasks, you will pass through various mazes. Along the way, you will encounter multiple intersections where you must decide whether to proceed straight, turn left, or turn right. You will take six trips through each maze. During the first trip, an arrow will guide you in the correct direction at each intersection. During the subsequent five trips, the arrows will be absent, and you have to indicate the direction yourself. To indicate hat direction, please move the joystick in the corresponding direction within a time frame of 3 seconds. You will then be informed whether your response was correct (O), incorrect (X), or late (?). Afterwars, regardless of your answer, you will be transported in the correct direction to the next intersection. At the end of the trip, you will see a trophy, followed by a short break, and you will then be transported back to the starting point for the next trip.”

Before maze S: “In this task, you will pass through a maze along a predetermined route where all intersections appear nearly identical. Therefore, it is important for you to memorize the sequence of directions within the labyrinth.”

Before maze A: “In this task, you will pass through a maze along a predetermined route. At each intersection, you will encounter photos that will provide you with information about the correct direction to take. For example, the photo of a garden might remind you to turn left at that particular intersection. The task is not easy because the order of the photos will change in each trip. For instance, the garden photo may appear at the third intersection of the first trial, but at the seventh intersection of the second trip. However, you should always turn left when you encounter the garden photo. It therefore is important for you to remember the relationship between the photos and the corresponding directions. Initially, there will be arrows provided as guidance during the run.”

Before maze B: “In this task, you will pass through a maze along the shortest possible route to reach a tree that is continuously visible in the distance. If you encounter a barrier along the way, it indicates that the corresponding turn is blocked. The tree is located directly outside the outer wall of the maze, while your destination is within the labyrinth, just inside the outer wall. On each trip, the tree will be positioned at a different location outside the maze, and your task is to always move towards that tree. You are currently at the starting point, with the tree visible to your left.”

Before maze R: “In this task, you will pass through a maze along the shortest possible route towards two object that are continuously visible in the distance: a tower and an electricity pole. If you encounter a barrier along the way, it indicates that the corresponding turn is blocked. The tower and the electricity pole are located directly outside the maze, while your destination is within the maze, where your distance from the tower and from the pole equals the distance between tower and pole. On each trip, the tower and the pole will be positioned at a different location outside the maze, and your task is to always move towards the location where you, tower and pole are equidistant. You are currently at the starting point and can see the tower on your left side.” [Because of software constraints, the pole appeared on the first trip only when reaching the second intersection.]

Before maze C: “In this task, you will visit photos in a maze according to a predetermined sequence. Here you see, from a bird’s-eye view, that the photos are located at every second intersection. [Top view of a grid maze is shown, with black lines representing the locations of photos.] Different photos are displayed at each intersection. To help you stay oriented, the outer walls of the maze are colored differently. At each intersection, you will see an above-head display of the next photo to visit, and your objective is to take the shortest route to the intersection where that particular photo is located. On your first trip, you will visit the photos row by row. [A meandring route is pointed out on the top view of the grid maze.] In the subsequent trips, however, that order will change. Nonetheless, each photos will be placed in the same locations on all trips. It is crucial for you to remember the locations of each photo within the maze.”

After the first trip through each maze: “You now are back at the starting point and have to follow the route without the help by arrows. There is no need to rush. If you are unsure about the correct direction, feel free to make a guess.”

After the second to fourth trip through each maze: “You now are back at the starting point. Please follow the route again.”

## Footnotes

1 Note that the term ‘decision making’ has a different meaning in literature on new route generation rather than route following, where it often refers, e.g., to the choice of a shortcut rather than of a familiar path.
